# Noninvasive high-frequency oscillatory ventilation as respiratory support in preterm infants: a meta-analysis of randomized controlled trials

**DOI:** 10.1186/s12931-019-1023-0

**Published:** 2019-03-15

**Authors:** Jing Li, Xiaoxia Li, Xianmei Huang, Zhiqun Zhang

**Affiliations:** Department of Neonatology, Affiliated Hangzhou First People’s Hospital, Zhejiang University School of Medicine, China, No. 261 Huansha Road, Hangzhou City, Zhejiang, 310002 China

**Keywords:** Noninvasive high-frequency oscillatory ventilation, Continuous positive airway pressure, Preterm infants, Bronchopulmonary dysplasia

## Abstract

**Background:**

Noninvasive high-frequency oscillatory ventilation (nHFOV), a relatively new modality, is gaining popularity despite scarce evidence. This meta-analysis was designed to evaluate the efficacy and safety of nHFOV as respiratory support in premature infants.

**Methods:**

We searched MEDLINE, EMBASE, CINAHL, and Cochrane CENTRAL from inception of the database to January 2019. All published randomized controlled trials (RCTs) evaluating the effect of nHFOV therapy with nasal continuous positive airway pressure (nCPAP) or biphasic nCPAP (BP-CPAP) in newborns for respiratory support were included. All meta-analyses were performed using Review Manager 5.3.

**Results:**

A total of 8 RCTs involving 463 patients were included. The meta-analysis estimated a lower risk of intubation (relative risk = 0.50, 95% confidence interval of 0.36 to 0.70) and more effective clearance of carbon dioxide (weighted mean difference = − 4.61, 95% confidence interval of − 7.94 to − 1.28) in the nHFOV group than in the nCPAP/BP-CPAP group.

**Conclusions:**

Our meta-analysis of RCTs suggests that nHFOV, as respiratory support in preterm infants, significantly remove carbon dioxide and reduce the risk of intubation compared with nCPAP/BP-CPAP. The appropriate parameter settings for different types of noninvasive high-frequency ventilators, the effect of nHFOV in extremely preterm infants, and the long-term safety of nHFOV need to be assessed in large trials.

## Background

Respiratory distress occurs in 7% of newborn infants and is increasingly common even in late preterm births [1]. Respiratory distress syndromes and infections are represented in approximately half of all cases of preterm infants [1]. Despite varied causes, the goals of managing respiratory distress include maintaining airway patency and providing respiratory support to deliver oxygen and remove carbon dioxide. In severe respiratory distress, these goals are often achieved through mechanical ventilation [2]. Invasive mechanical ventilation (IMV) increases survival in preterm infants with severe respiratory distress syndrome (RDS) [3]. However, IMV is associated with bronchopulmonary dysplasia (BPD) and impaired neurodevelopmental outcomes in preterm infants [3, 4]. Consequently, in recent years, several methods of noninvasive ventilation, including nasal continuous positive airway pressure (nCPAP), biphasic NCPAP (BP-CPAP), nasal intermittent positive-pressure ventilation, and high-flow nasal cannula, have been used with the hopes of preventing endotracheal mechanical ventilation and BPD [5]. Unfortunately, clinical studies have shown that up to 38–42% of very low birth weight infants experience treatment failure and require IMV [6, 7]. Moreover, although nCPAP is increasingly used, BPD rates have not declined [3, 8]. High-frequency ventilation is considered a gentler form of IMV with superior ventilation capability [9]. Applied noninvasive high-frequency oscillatory ventilation (nHFOV) may combine the benefits of nCPAP and high-frequency ventilation, which include the absence of ventilator-patient asynchrony and high efficacy in removing carbon dioxide (CO_2_) [10]. nHFOV is already used in European neonatal intensive care units despite scarce evidence to support the routine use of nHFOV [11]. Reviews of observational studies show an advantage with nHFOV for CO_2_ clearance in preterm infants treated for respiratory distress syndrome [12–14]. However, clinical trials could not demonstrate increased carbon dioxide clearance when applying nHFOV versus nCPAP [15, 16]. Because of the conflicting findings from reviews of observational studies and randomized trials, we have conducted a comprehensive systematic review evaluating all evidence by collecting data from randomized trials and prospective cohort studies.

## Methods

### Study identification and selection

This systematic review was conducted and is reported according to the recommendations of the Preferred Reporting Items for Systematic Reviews and Meta-Analyses (PRISMA) statement [17]. Electronic searches were performed in multiple databases, including PubMed, EMBASE, the Cochrane Controlled Trials Register, the Cochrane Library, Google Scholar, VIP, and Google, for relevant articles published from inception of the databases up to January 2019. The bibliographies of all potentially relevant articles were manually searched to identify any additional articles of relevance. No language restriction was applied. In addition, experts in the field were contacted to identify any ongoing or unpublished trials, although no studies were identified by this strategy. The protocol of this systematic review was registered before the literature search in PROSPERO (Prospero2016 CRD42016053475).

### Eligibility criteria

For inclusion, a study had to meet the following criteria: 1) it was a randomized controlled trial or crossover trial for evaluating interventions with a temporary effect; 2) preterm infants were randomized to receive respiratory support with nHFOV vs nCPAP/BP-CPAP; and 3) it reported more than one of the following outcome parameters: partial pressure of carbon dioxide (pCO_2_) levels, ΔpCO2 (variation difference of each group before crossover in randomized controlled crossover trials), and intubation. Exclusion criteria were as follows: a) non-clinical studies (experimental and basic studies); b) observational or retrospective studies; c) duplicate reports, secondary or post hoc analyses of the same study population; and d) studies with a lack of sufficient information on baseline, primary or secondary outcome data.

### Assessment of the risk of bias

Two reviewers (Zhang and Li) independently assessed the risk of bias of individual studies and the bias domains across studies using the Cochrane collaboration tool [18]. All discrepancies were resolved by discussion and consensus. The studies were rated to be at high risk of bias, low risk of bias, or unclear risk of bias based on sequence generation, concealment of allocation, blinding of participants/parents and personnel, blinding of outcome assessment, incomplete outcome data, and selective outcome reporting.

### Data collection

For each study, data were extracted independently by two reviewers (Zhang and Li) using a predesigned form. Any differences and disagreements in the collected data were discussed and resolved by consensus. Details of methodological quality, study design, analysis, and results were noted. For each outcome, the numeric results, the statistical methods used, and the *P* value were recorded. For randomized controlled crossover trials, because of carry-over, we only included data from the first stage for analysis. We contacted authors of the original reports to obtain further details when information regarding any of the above information was unclear.

### Statistical analysis

The statistical analyses were performed by the Mantel-Haenszel method (fixed-effect model) or the DerSimonian and Laird method (random-effect model) using the Review Manager meta-analysis software (version 5.3, 2012; The Cochrane Collaboration, Copenhagen, Denmark). Treatment effect estimates for all trials were calculated and expressed as typical relative risk (RR) for dichotomous outcomes and weighted mean difference (WMD) for continuous outcomes, all with a 95% confidence interval. The between-trial presence of heterogeneity among the recorded treatment effects was analysed by the χ2 test for heterogeneity and the I^2^ statistic, which expresses the proportion of heterogeneity that cannot be explained by chance. Heterogeneity was deemed significant when the corresponding *P* value was < 0.1 or when the I^2^ percentage was > 50, at which point the random-effect model was used. Otherwise, a fixed-effect model was applied [19]. Subgroup analyses or sensitivity analyses were carried out to assess the source of heterogeneity. When more than 10 articles were included, the presence of publication bias was assessed and displayed through a funnel plot.

## Results

### Study selection, description and assessment

**Fig. 1:**
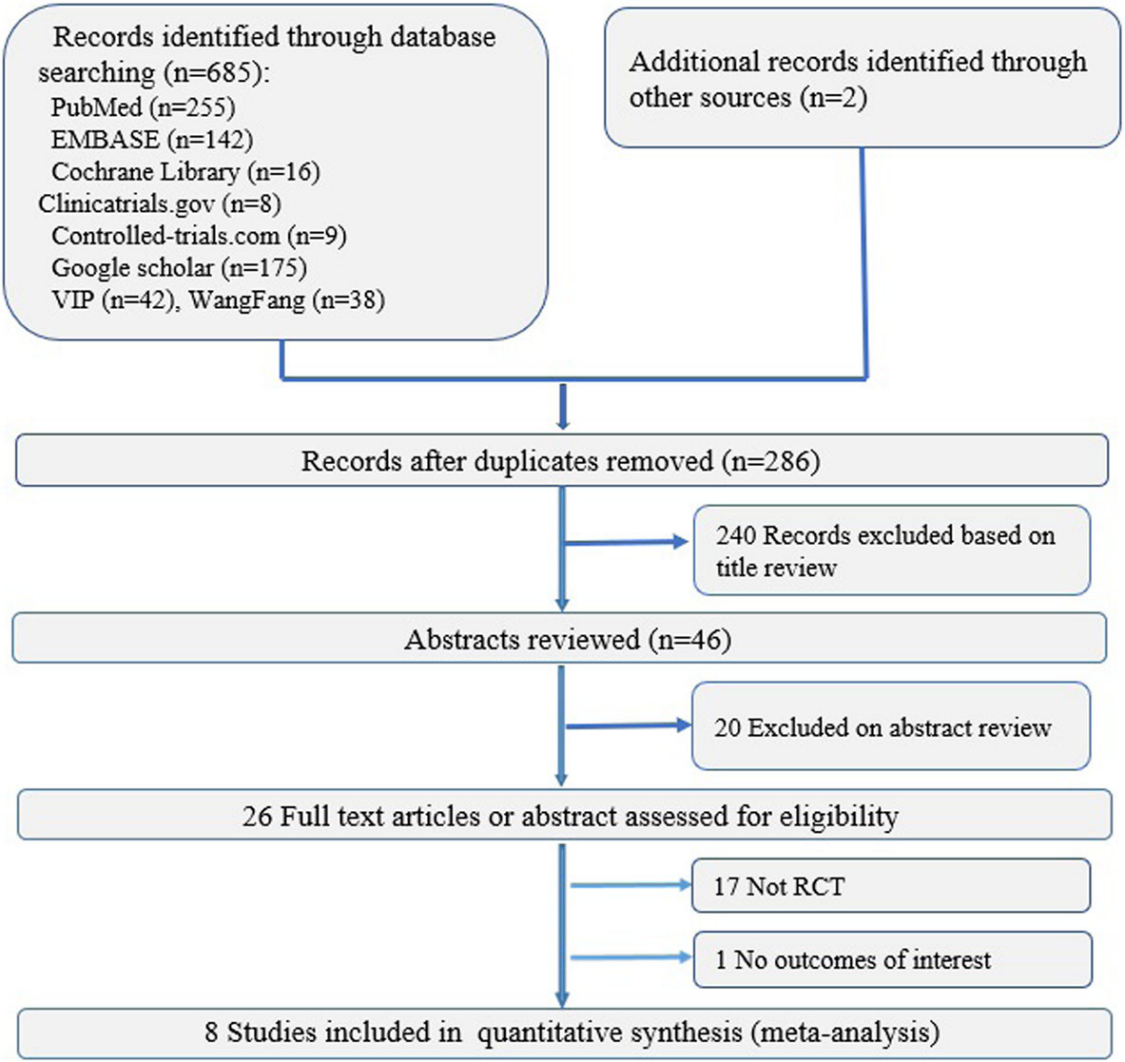
A PRISMA flow chart for the selection of eligible studies

The search strategy resulted in 286 potentially relevant citations. The PRISMA flow diagram (Fig. 1) summarizes the process of the literature search and study selection. After screening the titles and abstracts, we read 26 full-text articles or abstracts and assessed them for eligibility. Eight RCTs [16, 20–26] comprising 463 participants met the inclusion criteria. Overall, 6 trials [16, 20–22, 25, 26] of nHFOV vs nCPAP as respiratory support in preterm infants included 359 infants, and 2 trials [22, 24] of nHFOV vs BP-CPAP as respiratory support included 104 infants.

### Characteristics of the included studies

The 8 RCTs selected for analysis included a total of 463 participants (Tables 1, 2 and 3) [16, 20–26]. The publication dates of the RCTs ranged from 2016 to 2018. The nHFOV group vs nCPAP/BP-CPAP group were well-matched; the birth weight and gestational age did not differ significantly. Other aspects of respiratory treatment, including the resuscitation devices used and the criteria for using antenatal glucocorticoids as well as surfactant, were adequately described in the studies and conformed to current international guidelines. The incidence of neonatal respiratory distress syndrome (diagnosed based on respiratory symptoms and corresponding X-ray changes) was comparable between the nHFOV group and the nCPAP/BP-CPAP group.

**Table 1:** Characteristics of 8 RCTs and baseline characteristics of patients

Study	Study design	Group	N	Male (n)	GA (wk)	BW (g)	Antenatal steroid	Surfactant (n)	Caffeine (n)
Bottino 2018 [20]	Randomized controlled crossover trial	nHFOV	15	NA	< 32	< 1500	NA	15	15
		nCPAP	15	NA	< 32	< 1500	NA	15	15
Klotz 2017 [16]	Randomized controlled crossover trial	nHFOV	13	2	26.1 (2.2)*	814.2 (208.2)*	13	13	13
		nCPAP	13	9	27.2 (2.0)*	1083.5 (359.1)*	13	13	13
Lou 2017 [21]	Randomized controlled trial	nHFOV	34	24	32.5 (1.3)*	1790 (350)*	13	34	NA
		nCPAP	31	22	32.4 (1.4)*	1850 (410)*	12	21	NA
Lou 2018 [22]	Randomized controlled trial	nHFOV	33	18	33.5 (1.5)*	1790 (330)*	12	33	NA
		BPCPAP	32	17	34.2 (1.6)*	1840 (420)*	11	32	NA
Malakian 2018 [23]	Randomized controlled trial	nHFOV	63	28	31.08 (2.9)*	1485.5 (470)*	36	21	NA
		nCPAP	61	25	31.07 (2.8)*	1505.5 (490)*	31	23	NA
Mukerji 2017 [24]	Randomized controlled trial	nHFOV	16	1	26.1 (1.3)*	831.9 (150.1)*	12	10	NA
		BPCPAP	23	2	26.5 (1.6)*	878.0 (198.3)*	20	14	NA
Zhu 2017 [25]	Randomized controlled trial	nHFOV	37	22	31.7 (1.7)*	1670 (353)*	13	37	NA
		nCPAP	39	21	32.0 (1.9)*	1735 (327)*	15	39	NA
Zhu 2017 [26]	Randomized controlled trial	nHFOV	17	10	31.7 (1.7)*	1670 (353)*	6	17	NA
		nCPAP	21	12	32.0 (1.9)*	1735 (327)*	8	21	NA

*NA* Not Applicated, *BW*: Birth weight, *means ±SD

**Table 2:** Interventions used in the 7 RCTs

Study	Type of intervention	Ventilator type/ Interface	Ventilator parameter setting	Failure of intervention	Targeted SpO2
Bottino 2018 [20]	Primary respiratory support/ Following extubation	nHFOV: Medin, Olching, Germany; nCPAP: Interface: Short binasal prongs	nHFOV: Flow:7~10 L/min, Frequency: 10 Hz, Amplitude: set interval 10, I: E:1:1. nCPAP:	NA	90~95%
Klotz 2017 [16]	Backup ventilatory support/ Primary respiratory support	nHFOV/nCPAP: Sophie, Stephan, Gaggenbach, Germany; Leoni plus, Heinen+Löwenstein, Bad Ems, Germany Interface: binasal prongs or nasal masks	nHFOV: Frequency: 10 Hz, Amplitude: was set to achieve clearly visible oscillations of the chest, MAP: nHFOV and nCPAP were set at the level equal.	More than two episodes of apnea or bradycardia per hour; respiratory acidosis with a pH < 7.10; or FiO2 > 0.6 to maintain a SpO2 > 86%; hypercapnia with pCO2 > 70 mmHg.	86~96%
Lou 2017 [21]	Respiratory support after extubation	nHFOV: SLEbaby5000, Germany. nCPAP: Stephan, Germany. Interface: Short binasal prongs	nHFOV: FiO_2_: 0.35~0.40, Frequency: 6~12 Hz, MAP: 5~7 cmH2O, Amplitude: is 2 to 3 times that of MAP, specifically based on visible oscillations of the chest. nCPAP: FiO_2_: 0.30~0.40, PEEP: 4~6 cmH2O, Flow: 8~10 L/min.	More than 4 episodes of apnea per day, or saturation (SpO2) of < 85%, or paO_2_ < 50 mmHg, or pCO_2_ > 60 mm Hg.	90~95%
Lou 2018 [22]	Primary respiratory support	nHFOV: SLEbaby5000, Germany; BP-CPAP: Fabian, Swiss. Interface: Short binasal prongs	nHFOV: FiO_2_: 0.30~0.40, Frequency: 6~12 Hz, MAP: 6~12 cmH2O, Amplitude: is 2 to 3 times that of MAP, specifically based on visible oscillations of the chest. BP-CPAP: FiO_2_: 0.30~0.40, lower/higher PEEP: 5/12~15 cmH2O.	More than 4 episodes of apnea per day, or when FIO_2_ > 0.5, saturation (SpO2) of < 85%, or paO_2_ < 50 mmHg, or pCO_2_ > 60 mm Hg.	90~95%
Malakian 2018 [23]	Primary respiratory support	nHFOV: Medin, Olching, Germany; nCPAP: Infant Flow-driver device Interface: Short binasal prongs	nHFOV: MAP: 4~8 cmH_2_O, Frequency: 5 Hz, Amplitude: MAP: 3 cmH_2_O, FiO2:0.4~0.6. nCPAP: PEEP: 4~8 cmH2O, FiO2:0.4~0.6	at least one of the following: pH ≤7.20 and PaCO2 ≥ 60 mmHg, PaO2 ≤ 50 mmHg with a fraction of inspired oxygen of ≥0.6 or recurrent apnea with ≥3 episodes per hour associated with bradycardia, or a single episode of apnea that required bag-and-mask ventilation	≥ 90%
Mukerji 2017 [24]	Following CPAP failure	nHFOV: Drager VN500, Lubeck, Germany; BP-CPAP: SiPAP, Carefusion, USA. Interface: Short binasal prongs or nasal masks	nHFOV: FiO_2_: < 0.6, Frequency: 6~14 Hz, MAP: 8~10 cmH_2_O, Amplitude: were adjusted to achieve palpable/visible chest vibrations. BP-CPAP: FiO_2_: < 0.6, lower/higher PEEP: 5~7/8~10 cmH_2_O.	Intubation 7 days post randomization, Not specifically described.	90~95%
Zhu 2017 [25]	Primary respiratory support	nHFOV: Medin, Olching, Germany; nCPAP: Stephan, Germany Interface: Binasal prongs	nHFOV: Flow:8~12 L/min, Frequency: 6~12 Hz, Amplitude: 7~10. nCPAP: PEEP: 6 cmH2O.	PaCO2 > 60 mmHg with pH < 7.20, or more than 3 episodes of apnea per hour that required bag and mask ventilation, or hypoxia (FiO2 > 0.5 with PaO2 < 50 mmHg), or pulmonary hemorrhage.	90~94%
Zhu 2017 [26]	Primary respiratory support	nHFOV: Medin, Olching, Germany; nCPAP: Fabian, Swiss Interface: Binasal prongs	nHFOV: Flow:8~12 L/min, Frequency: 6~12 Hz, Amplitude: 6~10, FiO2:0.25~0.6. nCPAP: PEEP: 5~8 cmH2O, FiO_2_:0.25~0.6	FiO2 > 0.5 with PaO2 < 50 mmHg; PaCO2 > 60 mmHg with pH < 7.20; Frequent apnea episode (> 3/h)	88~93%

*nHFOV* noninvasive high-frequency oscillatory ventilation, *nCPAP* nasal continuous positive airway pressure, *BP-CPAP* biphasic continuous positive airway pressure, *MAP* Mean airway pressure, *PEEP* Positive end expiratory pressure, *FiO*_*2*_: fraction of inspired oxygen

**Table 3:** Outcomes measured in the 7 RCTs

Study	Group	pCO2 levels*	△pCO2*	Intubation
Bottino 2018 [20]	nHFOV	46.6 (7.5)	− 3.4 (7.77)	0/15
	nCPAP	49.9 (6.7)	1.4 (7.31)	0/15
Klotz 2017 [16]	nHFOV	54.8 (14.6)	3.6 (12.66)	0/13
	nCPAP	49 (8.1)	−1 (7.01)	0/13
Lou 2017 [21]	nHFOV	35.1 (7.8)	−1.4 (7.85)	5/34
	nCPAP	40.6 (7.8)	2.4 (7.24)	12/31
Lou 2018 [22]	nHFOV	41.5 (6.3)	−13 (7.43)	9/33
	BP-CPAP	50.5 (6.5)	−3.1 (7.0)	10/32
Malakian 2018 [23]	nHFOV			4/63
	nCPAP			9/61
Mukerji 2017 [24]	nHFOV	NA	NA	6/16
	BP-CPAP	NA	NA	15/23
Zhu 2017 [25]	nHFOV	NA	NA	9/37
	nCPAP	NA	NA	22/39
Zhu 2017 [26]	nHFOV	43.7 (5.6)	−12.1 (5.08)	4/17
	nCPAP	48 (4.7)	−6.3 (4.29)	12/21

*NA* Not Applicated, * means ± SD, pCO2 levels refers to Post nHFOV or nCPAP/BP-CPAP

### Risk of bias within individual studies

The risk of bias assessment for the included RCTs [16, 20–26] is reported in Table 4. Two trials were randomized controlled crossover trials [16, 20]. Most studies had a moderate to high risk of bias. Most bias stemmed from the blinding of the participants and personnel and the outcome assessments. The method of randomization was determined to be adequate in all studies. Four studies were found to have adequate concealment of allocation (Table 4).

**Table 4:** Risk of Bias Assessment for Included Randomized Clinical Trials

Source	Bias							
	Selection		Performance: Blinding of Participant and Personnel	Detection: Blinding of Outcome Assessment	Attrition: Incomplete Outcome Data	Selective Reporting	Other Sources	Overall
	Random Sequence Generation	Allocation Concealment						
Bottino 2018 [20]	Low risk	Low risk	High risk	High risk	Low risk	Low risk	Unclear risk	High risk
Klotz 2017 [16]	Low risk	Low risk	High risk	High risk	Low risk	Unclear risk	Unclear risk	High risk
Lou 2017 [21]	Low risk	Unclear risk	High risk	High risk	Low risk	Unclear risk	Unclear risk	High risk
Lou 2018 [22]	Low risk	Unclear risk	High risk	High risk	Low risk	Unclear risk	Unclear risk	High risk
Malakian 2018 [23]	Low risk	Unclear risk	High risk	High risk	High risk	Unclear risk	Unclear risk	High risk
Mukerji 2017 [24]	Low risk	Low risk	High risk	High risk	High risk	High risk	Unclear risk	High risk
Zhu 2017 [25]	Low risk	Low risk	Unclear risk	Unclear risk	Low risk	Unclear risk	Unclear risk	Moderate risk
Zhu 2017 [26]	Low risk	Unclear risk	Unclear risk	Unclear risk	Low risk	Unclear risk	Unclear risk	Moderate risk

### Systematic review of the findings from the collected results

**Fig. 2:**
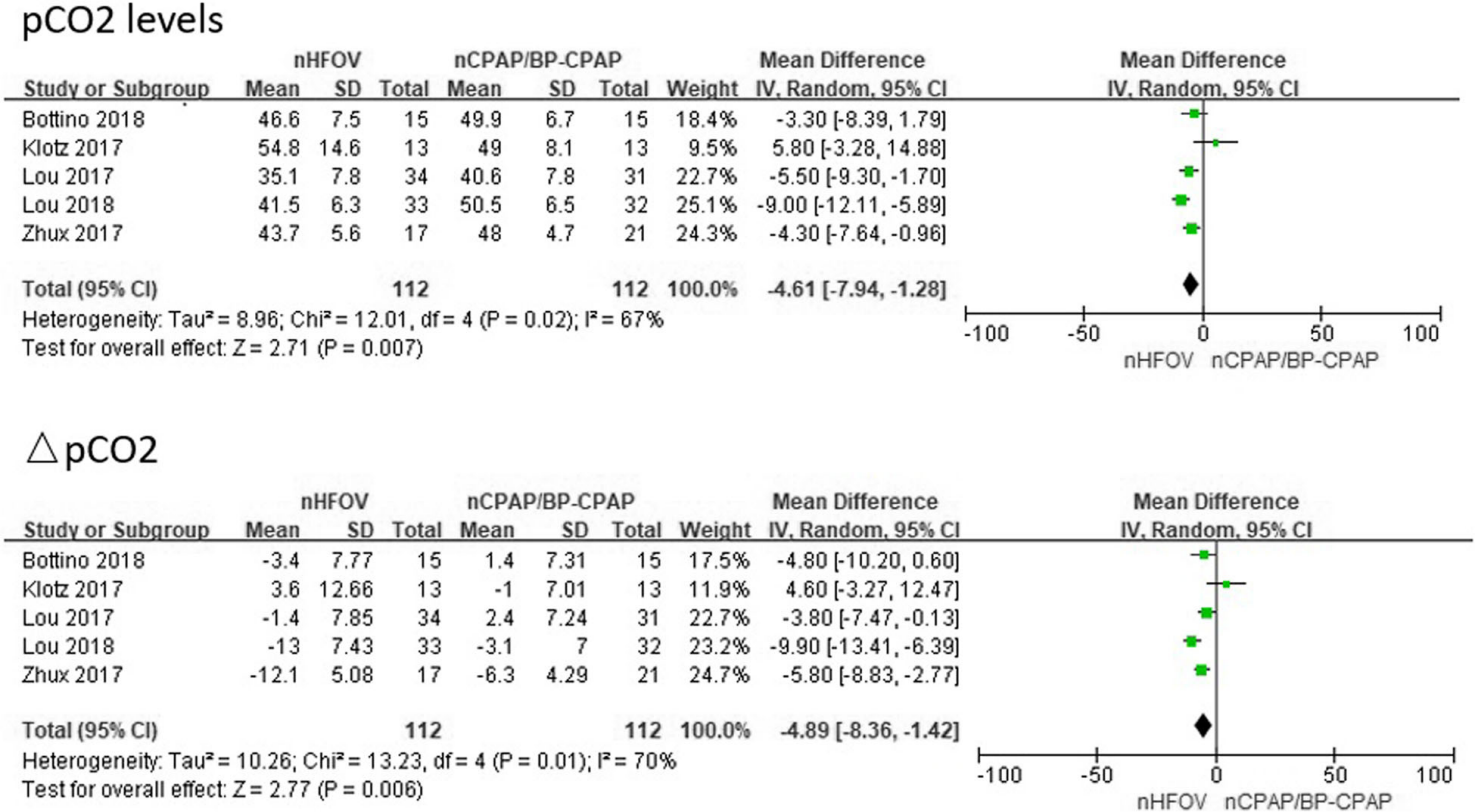
Meta-analysis of the effect of CO_2_ removal with the use of nHFOV or nCPAP/BP-CPAP

**Fig. 3:**
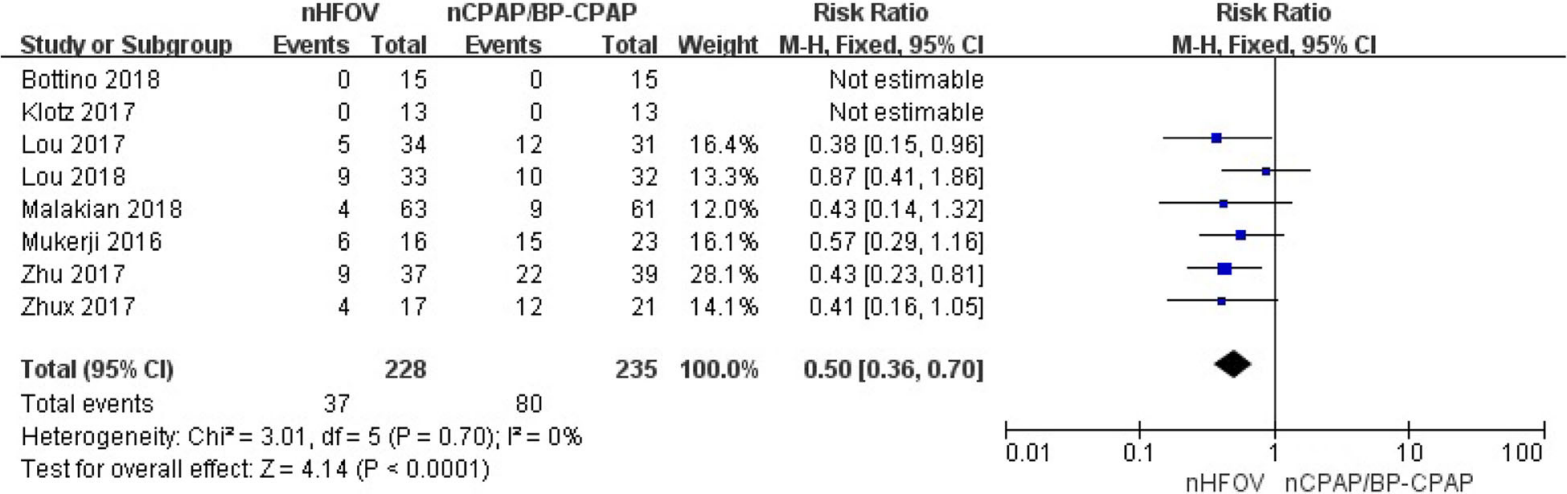
Meta-analysis of rate of intubation with the use of nHFOV or nCPAP/BP-CPAP

*pCO2 levels, ΔpCO2, and intubation.* Five trials enrolling 224 preterm infants reported pCO2 levels. Meta-analysis indicated that nHFOV significantly reduced pCO2 in preterm infants compared with nCPAP/BP-CPAP, including pCO2 levels (WMD = − 4.61, 95% CI -7.94 to − 1.28, I^2^ = 67%, *P* = 0.007) after respiratory support and ΔpCO2 (WMD = − 4.89, 95% CI -8.36 to − 1.42, I2 = 70%, *P* = 0.006) before and after respiratory support (Fig. 2). Eight trials enrolling 283 preterm infants reported on the rates of intubation. Meta-analysis indicated that nHFOV was associated with a lower likelihood of intubation for mechanical ventilation within 7 days than nCPAP/BP-CPAP was (RR = 0.50, 95% CI 0.36 to 0.70, I2 = 0%, *P* < 0.0001) (Fig. 3). A sensitivity analysis restricted to studies clearly stating the positive results [22, 25] showed similar results (RR = 0.57, 95% CI 0.38 to 0.87, I^2^ = 0%, *P* = 0.009).

## Discussion

This meta-analysis identified 8 randomized trials with 463 premature infants that compared respiratory support with nHFOV to that with nCPAP/BP-CPAP in preterm infants. The results of this systematic review of available RCTs show that in preterm infants, the use of nHFOV rather than nCPAP/BP-CPAP was beneficial in terms of improved CO_2_ elimination and a reduced risk of intubation for mechanical ventilation.

The quality of systematic reviews depends on the quality of the studies included. We evaluated the risk of bias in the RCTs analysed. Methodological issues may affect the study quality. We scrutinized the selected studies for good methodologic quality using strict quality assessment criteria [27]. The present review, to the best of our knowledge, is the first meta-analysis of RCTs reporting on the use of nHFOV compared with nCPAP/BP-CPAP as respiratory support in preterm infants. As a new noninvasive respiratory support method; there are relatively few prospective studies on nHFOV for preterm infants, and most studies are retrospective studies [12–14]. Most bench studies have clarified the effectiveness of nHFOV in promoting carbon dioxide removal from intrinsic mechanical properties [28–30], and nHFOV is superior to NIPPV in lung CO_2_ elimination [31]. Noninvasive interfaces affect tidal volume (Tv) and DCO_2_, and smaller cannulae result in lower tidal volume (Tv) reaching the distal airways and less CO_2_ elimination [28, 29]. Using lower frequency and higher amplitude in the nHFOV device increases Tv and promotes CO_2_ removal [30]. Similar to our meta-analysis, most retrospective studies found that nHFOV can effectively remove CO_2_. Van der Hoeven et al. [12] investigated the efficacy of nHFOV in a heterogeneous group of 21 infants with moderate respiratory insufficiency and showed that nHFOV was effective in reducing pCO_2_. Mukerji et al. [13] reported that nHFOV significantly reduces the occurrences of apnoeas, bradycardias, desaturations and CO_2_ levels compared with No nHFOV. nHFOV is effective in decreasing pCO_2_ in stable premature infants who require nasal CPAP support [14]. Recently, two RCTs by Rüegger et al. [15] (no specific data were reported) and Klotz et al. [16] showed that nHFOV fails to increase CO_2_ clearance compared with nCPAP in preterm infants; the former study mainly evaluated clinical symptom outcomes and included the paired difference in the combined number of episodes of desaturation and bradycardia during the 120-min period [15], and the latter study enrolled some patients who already had low CO_2_ [16]. Reducing CO_2_ levels is beneficial for severe hypercapnia, which may be harmful to maintaining normal CO_2_ levels [32]. Moreover, invasive neonatal ventilators (Sophie, Stephan, Gaggenbach, Germany; Leoni plus, Heinen+Löwenstein, Bad Ems, Germany) can be used to make an nHFOV mode through bi-nasal prongs or nasal masks with the frequency set to 10 Hz [16]. Experimental and clinical observational data using nHFOV devices other than the CNO device (Medin, CNO) suggest the need for much higher amplitudes at a frequency of 10 Hz, especially for low-compliant lung disease, to have a clinically relevant effect on pCO_2_ [33]. Compared with the dedicated noninvasive high-frequency generator (Medin, CNO), nondedicated high-frequency ventilators may be prone to air leakage or may not reach the set parameters in the airway due to lack of air leakage compensation because there is no pressure sensor. Wang et al. [34] reported that the effect of a nondedicated high-frequency ventilator (SLE5000) is similar to that of a dedicated high-frequency noninvasive generator (Medin, CNO) in clearing carbon dioxide and requiring intubation; however, the mean airway pressure of a nondedicated noninvasive high-frequency ventilator (SLE5000) (10(9,11)) is significantly higher than that of a dedicated high-frequency generator (Medin, CNO) (5(5,7)) [3]. In addition, a bench study showed that the optimal frequency for CO_2_ removal was 8 Hz [31]. In addition to the results from model studies and retrospective studies, our meta-analysis that was based on randomized controlled trials also confirmed that nHFOV can significantly remove CO_2_. Although removing CO_2_ is a weak outcome and is not directly related to major clinical outcomes, infants who fail to respond to nCPAP/BP-CPAP and retain CO_2_ can avoid intubation altogether with nHFOV. The role of nHFOV in removing carbon dioxide seems undisputed, and we should pay more attention to the effectiveness of nHFOV in different lung diseases and the long-term effects of nHFOV, such as BPD, IVH, NEC, infancy respiratory function and neurodevelopmental outcomes.

Although retrospective studies reported the feasibility of nHFOV in preventing intubation or facilitating extubation [13, 35], four RCTs did not show that noninvasive high-frequency ventilation had significant advantages in avoiding intubation compared with nCPAP/BP-CPAP [22–24, 26]. However, our meta-analysis showed that noninvasive high-frequency ventilation can reduce the risk for intubation compared with nCPAP/BP-CPAP, and the finding was robust to sensitivity analysis. It is important to note that when trials mixed patients of different mechanical risks, inappropriate parameter settings of nHFOV may be useless. Therefore, lung mechanics may vary in different patients and in different moments during the disease course, and this may require adjustments of various parameters [31]. Since CO_2_ elimination under nHFOV is provided in the upper airway dead-space [31], it is probably unnecessary to increase ΔP to achieve a visible chest oscillation for less severely ill infants (such as those affected by RDS or TTN) [33]. However, for patients with BPD or acute-on-chronic respiratory failure, NHFOV with real oscillators at high amplitudes is possibly useful to avoid invasive ventilation [33]. This factor deserves targeted trials, which are rare and difficult to perform. Fortunately, two multi-centre trials (NCT03181958, NCT03099694) are presently ongoing to draw objective conclusions. To optimize respiratory support for different patients and different moments during the disease course, a tool to help trial designers go through explanatory and pragmatic trials is necessary [36, 37]. In addition, the interface may affect the ventilation effect. In vitro studies show that devices with short double prongs had the lowest resistance to flow when nCPAP was used for respiratory support [38]. A bench and in vivo study showed that the amplitude of oscillation obviously decreases when the mask is used for high-frequency oscillatory ventilation [39]. Two studies included in our meta-analysis used nasal masks [16, 24]. This may have affected the ventilation, including CO_2_ removal and intubation, in those studies.

Although 6 RCTs included in our meta-analysis reported BPD and air leak, and 5 RCTs reported IVH, because the vast majority of trials examined are small, with cross-over design and not powered for these outcomes; the population included in the RCTs is generally late preterm infants, it may affect an outcome of BPD. It is inappropriate to perform meta-analysis of these outcomes. Therefore, we believe that a large multi-centre trial is urgently needed to confirm the effect of nHFOV in extremely preterm infants and the safety of nHFOV.

Admittedly, several limitations in our meta-analysis might have affected the interpretation of the findings. The analysed trials differed in their study design and clinical characteristics of the study participants. Two of the studies we included were randomized controlled crossover trials. Although the first period of a cross-over trial is in effect a parallel group comparison, use of data from only the first period will be biased if, as is likely, the decision to do so is based on a test of carry-over [18]. There was heterogeneity in the characteristics of the participants and interventions (including types and parameter settings of the noninvasive high-frequency ventilator) and a lack of a standardized assessment of intubation risk. Due to the mechanical characteristics of nHFOV, there are no trials reporting on the training of this respiratory support; thus, there is bias created by the different expertise levels of NICU personnel in using the technique. Furthermore, our meta-analysis was limited by the overall low quality of evidence and lack of robustness in higher quality trials. Publication bias could not be ruled out using a funnel plot due to a limited number of studies. Additionally, subgroup analyses based on gestational age or birth weight could not be performed due to the lack of individual patient data. Most premature infants included in this meta-analysis had a gestational age of over 30 weeks. For preterm infants under 30 weeks of age, who are severely affected by RDS and face a high risk of BPD, nHFOV may be beneficial [40]. Other possible biases include: the use of different interfaces across the studies are known to impact on mechanical efficiency of all types of noninvasive ventilation and even for nHFOV [33]; the measure of pCO2 by different methods (arterial, arterialised capillary and transcutaneous) in different studies may affect the results. Some studies may use venous CO2 and this should be avoided. In addition to sound design and adequate sample size, future research should not ignore populations of extremely premature infants, complex respiratory physiology and evaluating comfort [40, 41].

## Conclusion

In summary, the results of our meta-analysis of RCTs suggest that nHFOV, as respiratory support in preterm infants, significantly reduces the pCO_2_ level and risk for intubation compared with nCPAP/BP-CPAP. The appropriate parameter settings for different types of noninvasive high-frequency ventilators, the effect of nHFOV in extremely preterm infants, and the long-term outcome of nHFOV need to be assessed in large trials.

## Additional file

Additional file 1: Figure S1. Meta-analysis of adverse outcome with the use of nHFOV or nCPAP/BP-CPAP.

## Abbreviations

BP-CPAP: Biphasic continuous positive airway pressure
BPD: Bronchopulmonary dysplasia
CO_2_: Carbon dioxide
FiO2: Fraction of inspired oxygen
IMV: Invasive mechanical ventilation
IVH: Intraventricular hemorrhage
MAP: Mean airway pressure
nCPAP: Nasal continuous positive airway pressure
NEC: Necrotizing enterocolitis
nHFOV: Noninvasive high-frequency oscillatory ventilation
pCO_2_: Partial pressure of carbon dioxide
PEEP: Positive end expiratory pressure
PRISMA: Preferred Reporting Items for Systematic Reviews and Meta-Analyses
RCTs: Randomized controlled trials
RDS: Respiratory distress syndrome
RR: Relative risk
WMD: Weighted mean difference
